# Interaction Characterization of a Tyrosine Kinase Inhibitor Erlotinib with a Model Transport Protein in the Presence of Quercetin: A Drug–Protein and Drug–Drug Interaction Investigation Using Multi-Spectroscopic and Computational Approaches

**DOI:** 10.3390/molecules27041265

**Published:** 2022-02-14

**Authors:** Tanveer A. Wani, Mohammed M. Alanazi, Nawaf A. Alsaif, Ahmed H. Bakheit, Seema Zargar, Ommalhasan Mohammed Alsalami, Azmat Ali Khan

**Affiliations:** 1 Department of Pharmaceutical Chemistry, College of Pharmacy, King Saud University, P.O. Box 2457, Riyadh 11451, Saudi Arabia; mmalanazi@ksu.edu.sa (M.M.A.); nalsaif@ksu.edu.sa (N.A.A.); abakheit@ksu.edu.sa (A.H.B.); azkhan@ksu.edu.sa (A.A.K.); 2 Department of Biochemistry, College of Science, King Saud University, P.O. Box 22452, Riyadh 11451, Saudi Arabia; szargar@ksu.edu.sa (S.Z.); oalfageeh@ksu.edu.sa (O.M.A.)

**Keywords:** erlotinib, bovine serum albumin, fluorescence quenching, binding interaction, quercetin, competition

## Abstract

The interaction between erlotinib (ERL) and bovine serum albumin (BSA) was studied in the presence of quercetin (QUR), a flavonoid with antioxidant properties. Ligands bind to the transport protein BSA resulting in competition between different ligands and displacing a bound ligand, resulting in higher plasma concentrations. Therefore, various spectroscopic experiments were conducted in addition to in silico studies to evaluate the interaction behavior of the BSA-ERL system in the presence and absence of QUR. The quenching curve and binding constants values suggest competition between QUR and ERL to bind to BSA. The binding constant for the BSA-ERL system decreased from 2.07 × 10^4^ to 0.02 × 10^2^ in the presence of QUR. The interaction of ERL with BSA at Site II is ruled out based on the site marker studies. The suggested Site on BSA for interaction with ERL is Site I. Stability of the BSA-ERL system was established with molecular dynamic simulation studies for both Site I and Site III interaction. In addition, the analysis can significantly help evaluate the effect of various quercetin-containing foods and supplements during the ERL-treatment regimen. In vitro binding evaluation provides a cheaper alternative approach to investigate ligand-protein interaction before clinical studies.

## 1. Introduction

The most affluent blood protein present in vertebrates is serum albumin (S.A.) and it is mainly responsible for transporting both endogenous and exogenous molecules to their target sites. It is the principal constituent of the plasma proteins (≈60%) [1] and plays a significant role in the pharmacokinetics, pharmacodynamics, and toxicity of drugs [2]. The two most common serum albumin used as model transport proteins are BSA and HSA (human serum albumin). There is almost 75.6% sequence homology and similar ligand binding characteristics between the two. Therefore, BSA is commonly used as an alternative in protein–ligand binding studies because of its ready availability and cost-effectiveness [3,4]. The two most common ligand binding sites on BSA are Sudllow Site I and II positioned in subdomain IIA and IIIA [5,6,7].

Simultaneous administration of two or more drugs might lead to a competition between the two drugs to bind to a similar site present on the BSA. The conformation of the protein binding pockets can be altered on interaction with ligands, thereby influencing the binding of other ligands to the same binding pockets [8,9]. The therapeutic or adverse effects of the drug depend on the free fraction of the drug available in the plasma, and this property of drugs is taken into account when prescribing two or more drugs in a multidrug therapy due to possible competition at the plasma protein binding level [10,11].

Erlotinib (ERL), a tyrosine kinase inhibitor, has anticancer properties and is approved to treat non-small cell lung cancer. There is a high epidermal growth factor receptor (EGRF) expression in certain cancers, and ERL inhibits EGFR [12]. One of the mechanisms of action suggested for the ERL acts by disabling the phosphorylation ability of EGFR, thus inhibiting the signal transduction cascade leading to malignant cell apoptosis. Even though several mechanisms of action for ERL are proposed, the exact mechanism is yet unknown [13,14]. The binding of ERL to serum proteins from different species such as humans, mice, and rats are 92%, 95%, and 92%, respectively [15].

Fresh fruits and vegetables contain phenolic compounds such as flavonoids and tannins, and one such flavonoid is quercetin [16]. Quercetin (QUR) is also present in various food supplements and has antioxidant properties [17]. Quercetin has chemoprotective and radioprotective properties. In addition, it protects normal cells from the adverse effects of chemotherapy and radiotherapy [18]. The pharmacokinetics of quercetin is variable in humans due to their diet history, genetic polymorphism, and metabolic variation because of gut microbiota [19,20]. Enhancement in the antitumor activity of a breast cancer drug on concurrent use of QUR has been reported [21].

Quercetin is highly metabolized, and the amount of QUR in systemic circulation varies based on inter-individual pharmacokinetics and the source of QUR. In addition, although different organs metabolize quercetin, its transport to these organs is dependent on systemic circulation [20,22]. Thus, quercetin may interfere with the binding of the other concomitantly used drugs, impacting their pharmacokinetics and displacing them from their albumin binding sites. Such interference may lead to a toxic or a subtherapeutic response of the drug in the presence of QUR [23,24,25,26]. In addition, the pharmacokinetics of some drugs is affected by concurrent usage of QUR [7,23,25]. For example, a study reported the sudden death of experimental animals on the simultaneous use of QUR and digoxin [26].

The flavonoid QUR binds to Site I, subdomain IIA of BSA. Further, the interaction of QUR with other concomitantly used drugs has also been investigated [27,28,29,30,31]. In addition, possible competition between ERL and QUR to bind to BSA may affect the binding of ERL to BSA and lead to undesired effects of ERL [27,28,29,30,31,32,33,34]. Therefore, the current study investigated the ERL and BSA interaction with multispectroscopic and computational methods. Additionally, the impact of QUR was analyzed on the binding of ERL to BSA.

## 2. Materials and Methods

### 2.1. Chemicals

The chemicals used in the study ERL and QUR were procured from Weihua Pharma Co., Limited (Hangzhou, Zhejiang, China) and fatty acid-free BSA from Sigma-Aldrich (St. Louis, MO, USA). The phosphate buffer saline (PBS) pH-7 was prepared afresh to prepare the stock solution. QUR and ERL were dissolved in DMSO (dimethyl sulfoxide) followed by dilution with PBS. Millipore water (type-I) was used to prepare the stock solutions.

### 2.2. Fluorescence Measurements

The spectrofluorometer FP-8200 (JASCO, Hachioji, Tokyo, Japan) was used to measure the fluorescence spectra. The instrument had a quartz cell, and the slit width was 5 nm. The inner filter effects were corrected for the reabsorption of emitted light using the equation [35]:

$$F_{cor}=F_{obs}\times e^{(A_{ex}+A_{em})/2}$$

*F_cor_* and *F_obs_* are the corrected and observed fluorescence in the above equation, respectively, and *A_ex_* and *A_em_* are excitation and emission wavelengths absorbance values, respectively. The BSA spectra were recorded for the binary system, which consisted of BSA-ERL and BSA-QUR, and the ternary system consisted of BSA-QUR-ERL and BSA-ERL-QUR systems. For the measurement of spectra in the binary system, the concentration of BSA was held constant at 1.50 μM and the concentration of ERL was between 0.00–27.5 μM and QUR was between (0.00–35 μM) The spectra were recorded at three temperatures of 298, 303 and 310 K for BSA-ERL system and 298 K for BSA-QUR system. The concentration of BSA was 1.50 μM in the presence of ERL, and the spectra were recorded with ERL 0.00–27.50 μM in the presence of QUR 5.5 μM in the ternary system.

The site marker studies were undertaken to establish the binding Site for ERL with phenylbutazone for Site I and Ibuprofen for Site II as markers. The spectra for BSA were recorded with ERL in the presence of site markers phenylbutazone and ibuprofen.

### 2.3. U.V. Absorption Measurements

The UV-Vis absorbance spectra were recorded with Shimadzu 1800 (Kyoto, Japan). The spectra for BSA were recorded in the presence of ERL (0.00–15.00 μM) and a fixed concentration of BSA μM.

### 2.4. Synchronous Fluorescence Spectroscopic (SFS) Studies

The microenvironmental changes in the fluorophore residues were identified with the SFS technique. The changes in the amino acid residues Tyr (tyrosine) and Trp (tryptophan) can be observed at ∆λ = 15 nm and ∆λ = 60 nm. A shift in the emission spectra indicates changes in the polarity of amino acid residues around the fluorophore molecules.

### 2.5. Three-Dimensional (3D) Fluorescence Spectroscopy

Structural changes and conformational changes in the protein are identified with 3D fluorescence spectra. In addition, microenvironmental fluctuations in the vicinity of fluorophore residues lead to conformational or structural changes in the protein molecules. The BSA-ERL 3D spectra were matched to the 3D spectra of BSA to identify any such variations in the fluorophore microenvironment.

### 2.6. Molecular Docking and Molecular Dynamic Simulation (MDS)

The molecular docking was performed with bioinformatics tools MGL Tools [36], Auto Dock Vina [37], Molecular Operating Environment software (MOE), and Discovery Studio visualization tool. The PDB structure for the BSA (PDB ID: 6qs9) obtained from Protein Data Bank was used for the molecular docking [38]. The molecular docking was carried out for the three binding subdomains of BSA, and three runs were carried out for each binding Site. The top-scoring conformations were selected and analyzed for the interaction. MOE default parameters were used for docking analysis. The molecular dynamic simulation was carried out with NAMD 2.13 Suite (http://www.ks.uiuc.edu/Research/namd (accessed on 20 September 2021)) [39]. The ligand structures were immersed in the TIP3P water box. The charges neutralization was carried out by adding Na^+^ or Cl^−^ with their maximum concentration of 0.15 M. The minimization of the system was carried out in MOE. The default MOE parameters were used for the minimization, which included Amber10: EHT force field. Partial Charge on LL toms was calculated using Amber10: EHT force field. The system’s energy was minimized to an RMS gradient of 0.1 kcal/mol/A2. As per the MOE manual, an RMS of 0.1 is sufficient for structure minimization for molecular dynamic simulation. The minimized system was gradually heated. The temperature raised from 0 K to 310 K at an incremental level of 50 K for 100 ps and equilibrated at each temperature step followed by an unrestrained run of 20 ns. Constrained light bond with 2 fs time step. Long-range electrostatics were treated via the particle mesh Ewald method. The grid box formed had following parameters and dimensions: solvent molecules—26,824, 1.009 g/cm^3^; Space group—P1, Triclinic, 1, 1×; size—(105.8, 83.9, 102.2); cell shape—(90.0, 90.0, 90.0). The parameters RMSD and RMSF were obtained for the Protein BSA and the protein in the presence of ligand (BSA-ERL) systems. The trajectory was visualized and analyzed with VMD 1.9.3 tool.

## 3. Results and Discussion

### 3.1. Fluorescence Quenching and Enhancement

**Figure 1:**
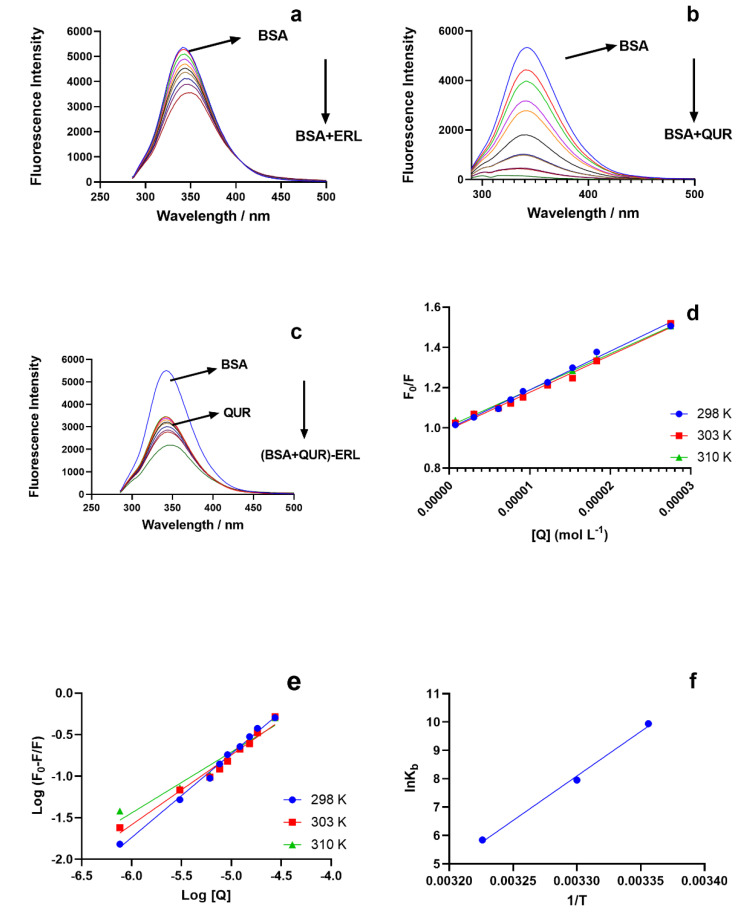
The fluorescence spectra for BSA (1.5 μM) with: (**a**) ERL (0.00–27.5 μM); (**b**) QUR (0.00–35 μM); (**c**) QUR (5.5 μM)-ERL (0.00–27.5 μM) at (λ_ex_ = 280 nm and λ_em_ = 300–500 nm); (**d**) Stern Volmer Plot for BSA(1.5 μM) -ERL (0.00–27.5 μM system at a temperature of 298, 303, 310 K; (**e**) double reciprocal plot [(*F*_0_ − *F*)/*F*] versus log [*Q*] for the BSA-ERL system to obtain the binding constant (**b**); (**f**) van’t Hoff plots to obtain the for thermodynamic parameters for the BSA-ERL system interaction.

The fluorescence spectra for the protein BSA for the binary and the ternary system were recorded at 280 nm (excitation wavelength) and an emission wavelength of 300–500 nm (Figure 1a–c). Both the ligands ERL and QUR decreased the fluorescence intensity of BSA. At increased ligand concentration ERL and QUR, the fluorescence intensity of BSA reduced further. The reduction in fluorescence intensity of BSA in the presence of QUR was higher than in the presence of ERL. A redshift of 8 nm was observed in the emission wavelength of BSA on interaction with ERL, indicating increased polarity and less hydrophilicity in the aromatic amino acid microenvironment [40,41,42]. A blue shift was also observed in the fluorescence emission spectra of BSA in the presence of QUR, as reported by earlier studies indicating higher hydrophobicity and a decline in polarity in the microenvironment of aromatic amino acid residues present in BSA [43]. The quenching behavior for the BSA-ERL binary system was determined with the help of the Stern Volmer equation [44]:

$$\frac{F_{0}}{F}=1+K_{sv}\left[Q\right]=1+k_{q}\tau_{0}\left[Q\right]$$

(1) $$k_{q}=K_{sv}/\tau_{0}$$

where *F*_0_ is BSA’s fluorescence intensity, and *F* is BSA’s fluorescence intensity in the presence of quencher. Stern Volmer constant is given as *K_sv_*_,_ and (Q) is quencher concentration. The *k_q_* biomolecular quenching constant and *τ*_0_ the lifetime of the fluorophore in the absence of quencher and is valued at 10^−8^ s for biopolymers.

The Stern Volmer plot for the BSA-ERL system is presented in Figure 1d. A decrease in the *K_sv_* values was observed with a rise in temperature for the BSA-ERL system (Table 1). Therefore, reducing *K_sv_* values with a temperature rise is associated with the static quenching mechanism. In the case of dynamic quenching, there is an increase in the *K_sv_* values [15,44]. Thus, a static quenching mechanism for the BSA-ERL system is suggested based on complex formation between BSA and ERL. The BSA-QUR system also follows a static quenching behavior as revealed by earlier studies [43]. The static quenching mechanism for the BSA-ERL system can also be established based on biomolecular quenching constants *k_q_*, which are of the order of 2 × 10^10^ L·mol^−1^s^−1^ for dynamic quenching and *k_q_* values higher than the maximum value of 2 × 10^10^ L·mol^−1^s^−1^ can be attained for biomolecular quenching constants only during a static quenching. Since the *k_q_* value for the BSA-ERL system given in (Table 1) was high, a static quenching mechanism is suggested for this system in accordance with earlier studies [15,44]. The quenching constant at room temperature for the (BSA-QUR)-ERL ternary system is presented in Table 2. The UV absorbance studies for the BSA-ERL system have reported an increase in the absorbance of BSA at 280 nm.

### 3.2. Binding Constant and Number of Binding Sites

The binding constants and binding stoichiometry are were determined using a double logarithmic regression plot derived from the equation: $\log\frac{\left(F_{0}-F\right)}{F}=\log\;K_{b}+n\;\log\left[Q\right]$.

*K_b_* and n represent the binding constant and binding stoichiometry in the above equation. The representative plot for the binding constants at the three studied temperatures of the binary system BSA-ERL is given in Figure 1e. The binding constant was obtained from the intercept of the double log plot, and the binding stoichiometry from its slope is presented in Table 2. Thus, the binding stoichiometry value of ≈1 suggests a single class of binding site was involved in the BSA-ERL interaction. The binding constants for the BSA-ERL system were of the order of 10^4^ M^−1^ suggesting a moderate binding [45]. The binding constant at room temperature determined for the BSA-QUR system was of the order of ≈(>10^6^). The ternary system (BSA-QUR)-ERL had binding constants of the order of ≈10^2^ (Table 2).

The binding constant for the BSA-ERL system was studied at various temperatures since the binding constants are temperature-dependent. Therefore, the thermodynamic processes involved in the BSA-ERL interaction were also investigated using the van’t Hoff equation and plot.

$$\ln K_{b}=-\frac{\Delta H{}^{\circ}}{RT}+\frac{\Delta S{}^{\circ}}{R}$$

ΔG°=ΔH°−TΔS°

In the equation above, Δ*H*° is enthalpy change, Δ*S*° is entropy change and Δ*G*° is Gibbs free energy, *R* is the universal gas constant, and *T* is the temperature in kelvins (K).

The thermodynamic parameters Δ*H°*, Δ*S°* and Δ*G*°, were calculated from the van’t Hoff plot for ln(*K_b_*) vs. 1/*T* and are given in Figure 1f.

All the three parameters, enthalpy change, entropy change, and Gibbs free energy, given in Table 2, attained negative values. A negative Gibbs free energy indicates a spontaneous interaction. Furthermore, the BSA-ERL interaction is suggested by van der Waals force and hydrogen bonds based on negative enthalpy and entropy. Since the enthalpy was −260 kJ·mol^−1^ whereas entropy was −0.79 kJ·mol^−1^, the BSA and ERL interaction indicate the interaction being enthalpy-driven, and the interaction had an unfavorable entropy.

### 3.3. Comparison of Binary and Ternary System Interactions

**Figure 2:**
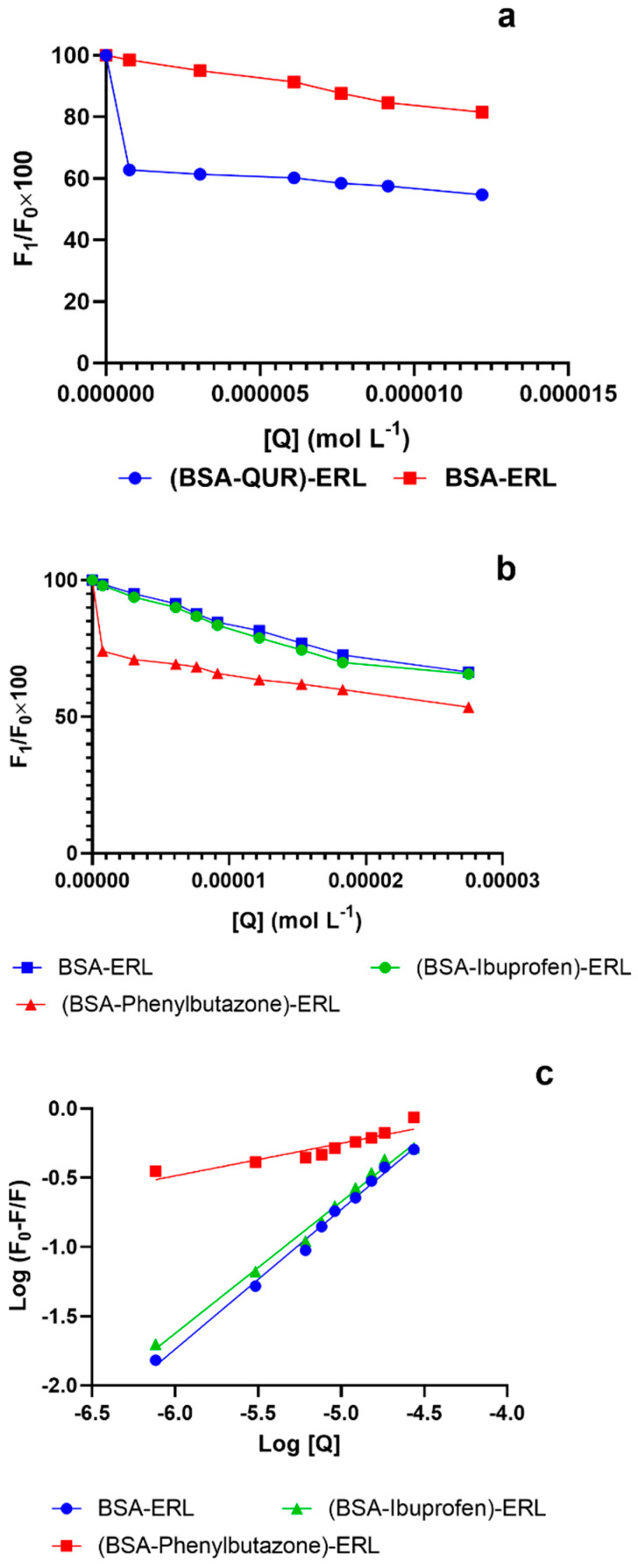
Quenching curve for BSA (1.5 μM)-ERL(0.00–27.5) (**a**); and BSA (1.5 μM)-ERL(0.00–27.5 μM) in presence of QUR (5.5 μM) (**b**); Quenching curve (**c**) and Binding constant for ERL in the presence of site markers phenylbutazone and ibuprofen.

In the BSA-ERL binary system, the quenching constants (Table 2) decreased at higher temperatures, suggesting the formation of a complex between BSA and ERL and a static quenching mechanism. Furthermore, the BSA-QUR system quenching mechanism was found to be in accordance with earlier studies [31,46] which reported a static quenching mechanism between them. For the ternary system (BSA-QUR)-ERL, the quenching constant in the presence of QUR for the BSA-ERL system was higher than in its absence. The rise of the quenching constant of the BSA-ERL system can be attributed to the fact that in the presence of QUR, the accessibility of ERL to BSA increases, improving the quenching efficacy of ERL. An earlier study reported a similar phenomenon whereby the quenching efficiency of gliclazide increased in the presence of QUR [43]. The fluorescence quenching plot for the binary system and the ternary system is provided in Figure 2a. In the BSA-ERL system, ERL reduced the fluorescence intensity of BSA by 19%. Whereas in the (BSA-QUR)-ERL system, the fluorescence intensity of BSA reduction was by almost 45%. Therefore, the presence of QUR in the BSA-ERL system further reduced the fluorescence intensity of the BSA-ERL system by 26%. The decrease in the fluorescence intensity of the BSA-ERL system suggests a strong influence of QUR in the BSA-ERL interaction.

The BSA-ERL binary system’s binding constant of ≈ 10^4^ suggests moderate binding between the ERL and BSA. Our results corroborated earlier studies for the interaction between BSA and ERL [15]. The binding constants for the BSA-ERL system lowered with a rise in temperature (Table 2). In the other binding system that consisted of BSA-QUR, it was observed that QUR had a strong binding interaction with BSA. Some studies have reported a strong binding interaction between QUR and serum albumin [43,47]. As a result of this interaction between QUR and BSA, ERL, which is moderately bound to BSA, may not displace the bound QUR from the binding sites present on BSA. Therefore, a ternary system was developed whereby the interaction between BSA and ERL was studied in the presence of QUR. Finally, it was concluded that QUR considerably affected the BSA-ERL system, and the presence of QUR caused a decrease in the binding constant of the BSA-ERL system from 2.0 × 10^4^ to 0.2 × 10^2^. (Table 2).

The binding Site for ERL was identified using phenylbutazone and ibuprofen as site-specific markers for Site I and Site II of BSA, respectively. Quenching curve and binding constants of the BSA-ERL system in the presence of site markers were compared to the binding constant of the BSA-ERL system in the absence of site markers. Ibuprofen presence did not influence the quenching behavior of the BSA-ERL system (Figure 2b), and binding constants in the presence and absence of ibuprofen were similar (Table 2). Thus, these results rule out the binding of ERL to BSA Site II. However, the presence of phenylbutazone in the BSA-ERL system strongly influenced the quenching behavior and the binding constant of the BSA-ERL system (Figure 2c). Hence, it was concluded that ERL binds to Site I of BSA. Furthermore, since QUR also binds to Site I of BSA [43,47], the presence of QUR in the BSA-ERL system can markedly influence the interaction between the BSA-ERL system.

Furthermore, QUR in the BSA-ERL system reduced the binding constants of the system, implying that the free drug fraction of ERL may rise in the systemic circulation in the presence of QUR. A study conducted in non-small cell lung cancer patients taking ERL reported that adherence to the treatment regimen of ERL depends on the severity of side effects in these patients [48]. It also reported that side effects were more severe in patients with higher plasma area under the curve (AUC) for ERL. One of the most common adverse events related to the ERL treatment regimen is skin disorders, including acneiform rash, xeroderma (dry skin), pruritus, and paronychia. Sometimes, these side effects are so severe and necessitate treatment interruption or cessation [49]. One of the reasons attributed to the severity of these side effects for ERL is its plasma concentrations. Therefore, the severity of side effects influences patients’ adherence to the treatment regimen. Since QUR may affect the AUC of ERL in plasma, increasing the plasma concentrations of ERL which might lead to unwanted adverse events affecting patients’ adherence to the treatment regimen.

### 3.4. UV Absorption Studies

**Figure 3:**
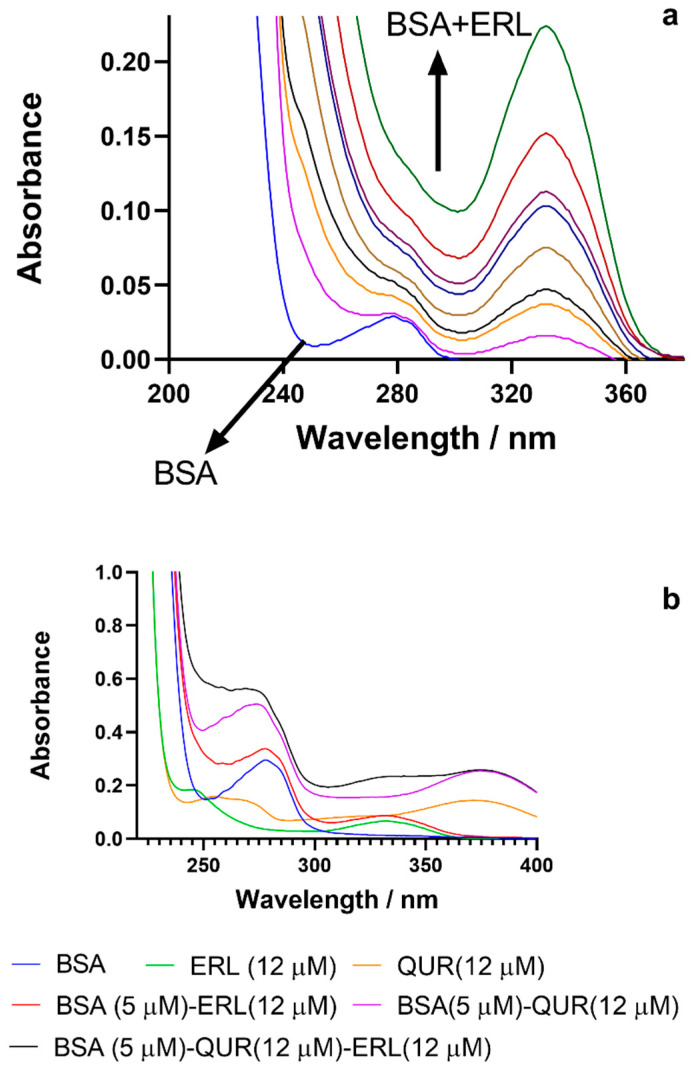
Ultraviolet–visible spectra for BSA (1.5 μM) in presence and absence of ERL (0.00–27.5 μM) (**a**); UV-visible spectra comparison for BSA, ERL, BSA-ERL, QUR, BSA-QUR, and BSA-ERL in the presence of QUR (**b**).

The two critical aspects that are studied by UV absorption are changes in the protein structure and protein–ligand complex formation [50]. The absorption spectral changes for BSA occur in protein–ligand complex formation and therefore infers a static quenching mechanism, whereas no spectral changes will be recorded in dynamic quenching [51]. Furthermore, the intensity of the BSA absorption peak at 280 nm was higher on interaction with ERL (Figure 3a), suggesting a static quenching mechanism between BSA and ERL.

Spectra for BSA was compared to spectra of the studied ligands ERL and QUR, BSA-ERL complex, and BSA-QUR complex. An increase in absorption spectra was observed for the BSA-ERL system compared to BSA alone. The ERL showed different absorption peaks compared to both BSA and BSA-ERL systems. The higher absorption peak for the BSA-QUR system was observed compared to BSA alone, and the spectra were different from the QUR absorption spectra.

The absorption spectra for the ternary system (BSA-QUR)-ERL was also studied, and it was different from both the binary system spectra (Figure 3b).

### 3.5. Synchronous Fluorescence Spectroscopic Studies

The microenvironment alterations in the fluorophore residues can be identified with synchronous fluorescence spectroscopy. The changes in emission wavelength indicate polarity change in the fluorophore residue microenvironment [52]. The synchronous spectra recorded at ∆λ = 15 nm and ∆λ = 60 nm provide information about the Tyr and Trp residue microenvironment. The spectra for Tyr showed no shift in the emission wavelength, whereas the spectra for Trp showed a slight 1 nm shift. A blue-shift at ∆λ = 60 nm suggests micro-environmental modifications in the vicinity of Trp residue. Further, it is recommended that the BSA experienced an increased hydrophobicity on interaction with ERL.

### 3.6. Three Dimensional (3D) Fluorescence Spectroscopy

**Figure 4:**
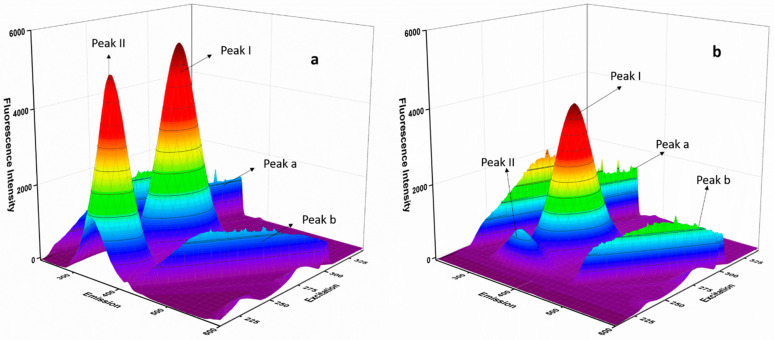
Three-dimensional fluorescence spectra for BSA (**a**) and BSA-ERL system (**b**).

The 3D fluorescence spectroscopy provides information about protein structural alteration on interaction with a ligand [41]. The 3D fluorescence spectra were accessed for the protein BSA and the protein–ligand BSA-ERL system (Figure 4a,b). Four peaks observed in the 3D spectra were identified as Peak a and b representing Rayleigh scattering (λ_ex_ = λ_em_) and IInd order scattering peak (λ_em_ = 2_ex_). Higher intensity and scattering were observed for Peak a in the BSA-ERL system compared to BSA since the BSA-ERL complex formed a bigger macromolecule than BSA. The other peaks, Peak I representing Trp and Tyr fluorophore residues and Peak II (polypeptide backbone structures), were evaluated in the 3D spectra. The intensity of both peaks, Peak I and Peak II, declined in the BSA-ERL protein–ligand system compared to the protein BSA. Hence, it is concluded that the interaction of ERL with BSA altered the microenvironment of the fluorophore residues, which led to these changes in the 3D spectra. Hence it was concluded that structural changes might have occurred in BSA on interaction with ERL.

### 3.7. Molecular Docking and Molecular Dynamic Simulation (MDS)

Molecular docking studies confirmed the experimental results for the BSA-ERL interaction. The site probe experiments with phenylbutazone suggest that the ERL interacted with BSA’s subdomain IIA (Site I). However, an earlier study using molecular docking suggested that ERL binds to subdomain IB (Site III) and subdomain IIIA (site II) of BSA [53]. Furthermore, the site probe study with ibuprofen (Site II marker) did not suggest the interaction of ERL at Site II of BSA. Moreover, the quenching curves for the BSA with ERL in the presence or absence of ibuprofen did not change, suggesting no interaction of ERL with site II of BSA.

**Figure 5:**
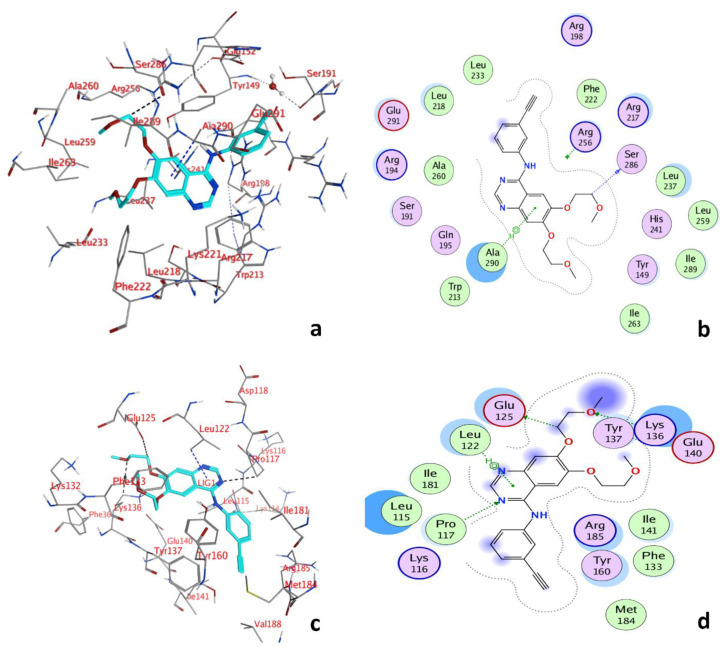
Two-dimensional molecular docking conformation for BSA ERL system at Site I (**a**) and Site III (**c**); three-dimensional docking conformation of BSA-ERL system Site I (**b**) and Site III (**d**).

Molecular docking conformation for the interaction of ERL to Site I and Site III of BSA is given in Figure 5a,c. The binding energy for Site I −33.63 kJ·mol^−1^ was lower than the binding energy −29.91 kJ·mol^−1^ for Site III of BSA. The binding pocket of Site I of BSA (Figure 5b) was surrounded by Glu291, Leu218, Leu233, Arg198, Phe222, Arg256, Arg217, Ser286, Leu237, Leu259, His241, Ile289, Tyr149, Ile263, Ala290, Trp213, Gln195, Ser191, Ala260, Arg194, and the amino acids that surrounded the binding pocket at Site III were Lys 116, Pro117, Leu115, Ile181, Leu122, Glu125, Tyr137, Lys136, Arg185, Ile141, Tyr160, Phe133, Met184. Furthermore, the fluorophore residues Trp213, Tyr149, and Phe222 were found in the vicinity of the binding pocket. Thus, the interaction of ERL with BSA might influence these fluorophore residues. In addition, the hydrogen bond between Ligand C19 and Ser286, 3.41 Å, and pi hydrogen bond between 6-ring and Ala290, 3.79 Å were also observed in the BSA-ERL interaction. The BSA-ERL interaction was also investigated for any changes in the presence of QUR. In this study, three hydrogen bonds, Ligand C15 and Phe133, 2.95 Å, Ligand O3 and Phe133, 2.65 Å, Ligand O4, and Lys136, 3.08 Å, were found. In addition, two pi-H bonds 6-ring and Leu115, 4.16 Å, 6-ring and Leu122, 3.90 Å, were observed.

At Site III (Figure 5d), the BSA and ERL formed three hydrogen bonds and one pi–hydrogen bond. The three hydrogen bonds formed were between Ligand C17 and Glu125, 3.51 Å, Ligand O4 and Lys136, 3.15 Å, Ligand N7, and PRO117, 3.15 Å, and the one pi-hydrogen bond formed was between 6-ring and Leu122, 3.79 Å.

As reported by earlier studies, QUR also binds to site I of serum albumin [47], and thus competition for the same binding Site might occur. Therefore, the molecular docking and the experimental results concluded that the presence of QUR might influence the binding of ERL to BSA.

**Figure 6:**
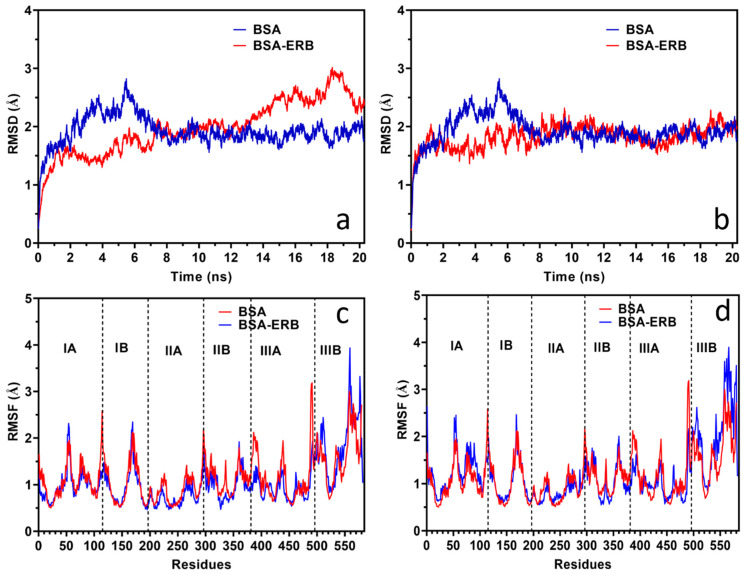
Molecular dynamic simulation RMSD plot for Site I (**a**) and Site III (**b**) and RMSF plot for Site I (**c**) and Site III (**d**).

The conformation stability for the BSA-ERL system was studied with MDS. The MDS study was conducted for Site I and Site III of BSA with ERL. The complex’s stability was evaluated based on the root mean square deviation (RMSD) and root mean square fluctuation (RMSF) studies. The RMSD studies for BSA and BSA Site I and Site III with ERL are provided in Figure 6a,b, respectively, whereas the RMSF plots are given in Figure 6c,d, respectively. The most critical deviations from the crystal structure are found at the residue level in the most mobile parts of the protein, i.e., loops, terminal regions, and helix ends. In contrast, the transmembrane segments remain stable in all the simulations.

Figure 6a shows the RMSD per residue for all the simulations reported in the present work for Site I. Three major peaks in subdomain 1 (loops C2) Ala78, Pro110, and Cys168 on the N-terminus were observed.

Further, in the simulation of the complex and comparison to the native protein residue Cys168 and Ala78, the N-terminus seems to be responsible for the higher RMSD values from 14–20 ns. Additional minor peaks can also be observed in the remaining loops 299, 309, and 504 and the C-terminus.

In the simulations between 2 to 8 ns, the resulting RMSD plots seem to be responsible for the higher RMSD values beyond residue Pro110 on the N-terminus for the native protein. No fluctuation was observed in the RMSD or RMSF plots of the BSA-ERL system at either of the binding sites, Site I or Site III. The RMSD averages were 1.98 and 1.82 Å, respectively, and the variation in the RMSD for the BSA-ERL system was between 0.258–3.013 Å for Site I and 0.214–2.318 Å for Site III. Therefore, a stable complex between BSA and ERL is concluded as the RMSD values did not fluctuate too high. Further, the residual flexibility is interpreted from the RMSF studies. The RMSF plot for both the studied sites (Site I and Site III) of the BSA-ERL system suggests that the complex formed between BSA and ERL was stable with a fluctuation of less than 3 Å [54].

## 4. Conclusions

This study examined the influence of flavonoid QUR on the BSA-ERL interaction by multispectroscopic and computational methods. Quercetin presence in the BSA-ERL system reduced the binding constant of the BSA-ERL system to almost half of what was observed in its absence. Thus, it can be concluded that there will be a higher free drug fraction of ERL in the system in the presence of QUR. However, the use of ERL in a therapeutic regimen leads to several adverse events, which in turn are associated with ERL plasma concentrations. Thus, co-administration of QUR and ERL might influence the pharmacokinetics of ERL and needs to be investigated by in vivo studies. Further, QUR is highly metabolized in the human body, necessitating studying the effect of the QUR on co-administered drugs in future studies. Hence, the information gained from such studies can benefit from dose optimization where the two drugs are intended to be co-administered.

## Figures and Tables

**Table 1:** Stern Volmer *K_sv_* and bimolecular quenching constant *k_q_*.

System	*T* (K)	*R*	*K_sv_* ± SD * (M^−1^)	*k_q_* × 10^12^ (M^−1^S^−1^)
BSA-ERL	298	0.9915	19,255.84 ± 625	1.93
	303	0.9887	18,450.98 ± 542	1.85
	307	0.9956	18,008.45 ± 489	1.80
BSA-Quercetin	298	0.9874	460,702 ± 1875	46.07
(BSA-QUR)-ERL	298	0.9954	26,970 ± 105	2.70

* standard deviation.

**Table 2:** Binding parameters binary and ternary systems and thermodynamic parameters for BSA-ERL system.

System	*T* (K)	*K_b_* ± SD *	*n*	∆*G*° (kJ·mol^−1^)	∆*H*° (kJ·mol^−1^)	∆*S*° (J mol^−1^·K^−1^)
BSA-ERL	298	2.07 ± 0.11 × 10^4^	1.01	−24.40	−260.80	−793.31
	303	2.83 ± 0.08 × 10^3^	0.84	−20.43		
	307	3.44 ± 0.04 × 10^2^	0.73	−17.26		
BSA-QUR	298	6.33 ± 0.07 × 10^6^	-	-	-	-
(BSA-QUR)-ERL	298	0.20 ± 0.05 × 10^2^	-	-	-	-

* standard deviation.

## Data Availability

Data will be available on request to corresponding author.

## Sample Availability

Samples are to be prepared freshly for analysis.
